# First Genome Sequence of Shallot Latent Carlavirus from *Allium macrostemon* Bunge

**DOI:** 10.1128/genomeA.00809-17

**Published:** 2017-08-17

**Authors:** Kazusato Ohshima, Kouta Okamura, Ryosuke Yasaka, Shinji Fukuda, Kanji Ishimaru, Yasuhiro Tomitaka, Kazuo Yamashita

**Affiliations:** a Department of Applied Biological Sciences, Faculty of Agriculture, Saga University, Saga, Japan; b The United Graduate School of Agricultural Sciences, Kagoshima University, Kagoshima, Japan; c Institute of Wild Onion Science, Saga University, Saga, Japan; d Saga University Center for Education and Research in Agricultural Innovation, Faculty of Agriculture, Saga University, Saga, Japan; e Kyushu Okinawa Agricultural Research Center, National Agriculture and Food Research Organization, Koshi, Japan; f Aomori Prefectural Industrial Technology Research Center, Aomori, Japan

## Abstract

A wild Japanese garlic plant (*Allium macrostemon* Bunge, wild onion) with leaves showing chlorotic stripes was collected in Saitama Prefecture, Japan. Genome sequencing showed that it was infected with shallot latent carlavirus. The genomic sequence of this virus is reported for the first time from wild onion.

## GENOME ANNOUNCEMENT

Wild Japanese garlic (*Allium macrostemon* Bunge) is the most widely distributed wild onion plant in Japan. Only scallion mosaic potyvirus has previously been reported from Japanese garlic (1). In the spring of 2013, a Japanese garlic with chlorotic striped leaves (plant no. WOST87; wild onion Saitama 87) was collected from a riverbank in Saitama Prefecture, Japan. We report here the genome sequence of *Shallot latent virus* (SLV) found in this plant and compare it to those of SLV isolates reported in earlier studies (2, 3). SLV is a species of the genus *Carlavirus* in the family *Betaflexiviridae* (4).

Sap from specimen WOST87 was manually inoculated to leaves of *Chenopodium quinoa* test plants, and chlorotic local lesions appeared on them. Viruses known to infect *Allium* spp. and to cause local lesions in *C. quinoa* were cucumber mosaic cucumovirus, tomato spotted wilt tospovirus, tobacco mosaic tobamovirus, and shallot latent carlavirus. Therefore, tests using enzyme-linked immunosorbent assays or reverse transcription-PCR (RT-PCR) (PrimeScript II high-fidelity one-step RT-PCR kit; Takara Bio, Shiga, Japan) were used to identify the virus. A part of the SLV genome was identified by RT-PCR when SLV-specific primer pairs with a NotI restriction site were used. These primer pairs amplify the region from the middle of the TGB1 coding region to the 3′ end of SLV genome. The RT-PCR products were cloned into the NotI site of the plasmid pZErO-2 vector. The nucleotide sequences of the region in three plasmid clones were determined, and this confirmed that the sequences were nearly identical to those reported for SLV. Therefore, we focused on this virus in the subsequent experiments.

The cDNAs of the remainder of the genome (2,303 nucleotides [nt], 3,705 nt, and 1,440 nt in length) were ampliﬁed and cloned similarly. The nucleotide sequences of at least three plasmid clones were determined for each region. The clone sequences with more than 400-bp overlapping regions were assembled to obtain the whole-genome sequence. The RT-PCR fragments were also directly sequenced. The whole-genome sequence, excluding a 23-nt 5′-end primer sequence used for amplifying the genome, was 8,338 nt in length.

The nucleotide and amino acid identities for the RNA-dependent RNA polymerase (RdRp) and coat protein (CP) coding regions of WOST87 and SLV (GenBank accession numbers NC_003557, JF320811, JQ899443, and HQ258896), were calculated using EMBOSS Needle (5). The nucleotide and amino acid identities in the RdRp coding region were from 74.9 to 75.2% and 84.6 to 85.7%, and those in the CP coding region were 78.4 to 79.2% and 92.9 to 94.6%, respectively. According to the species demarcation criteria given in the Ninth Report of the International Committee on Taxonomy of Viruses (ICTV), distinct species in the genus *Carlavirus* should have less than about 72% shared nucleotide identity (or 80% amino acid identity) in their RdRp and CP coding regions. Hence, our virus is an isolate of SLV, although, as its sequence identities were so different from those of previously reported SLV isolates, we consider our virus to be a distinct strain. This is the ﬁrst report of SLV collected from wild onion in Japan.

### Accession number(s).

The sequence has been deposited in GenBank/EMBL/DDBJ under the accession number LC279526.
